# The preface: physical properties and functional behaviours of metal halide perovskites and their derivatives

**DOI:** 10.1093/nsr/nwaf167

**Published:** 2025-04-28

**Authors:** Feng Gao, Samuel D Stranks

**Affiliations:** Department of Physics, Chemistry, and Biology (IFM), Linköping University, Sweden; Department of Chemical Engineering and Biotechnology, University of Cambridge, UK

## Abstract

This special issue brings together contributions from leading experts to explore the physical properties and novel functionalities of 3D halide perovskites, 2D halide perovskites, and organic-inorganic metal halides.

Metal halide perovskites represent a unique class of highly promising semiconductors that have attracted substantial attention in recent years. Thanks to their excellent photophysical properties and ease of solution processing, they have found widespread applications in solar cells, light-emitting diodes (LEDs), photodetectors, lasers and X-ray detectors [1–3]. Structurally, perovskites crystallize in a lattice composed of corner-sharing BX₆ octahedra, typically forming a 3D network with the general formula ABX₃. Beyond the 3D halide perovskites, modifications to the connectivity of the BX₆ octahedra have led to the formation of 2D perovskites and organic–inorganic metal halides (0D) [4]. These lower-dimensional derivatives also exhibit intriguing and tunable optical and electronic properties, further enriching the field.

In this Special Topic, we bring together contributions from leading experts to explore the physical properties and novel functionalities of 3D halide perovskites, 2D halide perovskites and organic–inorganic metal halides. The Special Topic includes one comprehensive review, one perspective article and four original research papers, offering both foundational insights and the latest advancements.

The review article by Zhang, Fu and co-workers provides a comprehensive overview of the roles of self-assembled monolayers (SAMs) in perovskite devices [5]. These layers serve as interfacial modifiers on both the bottom and top sides of the perovskite absorber, as well as transport layers. The review covers their evolution, molecular design and physicochemical properties, and discusses their role in improving charge transport, wettability, interfacial stress mitigation, mechanical strength and chemical stability. The review concludes with a thoughtful discussion on the rational design of SAMs and future challenges in their integration.

The perspective article by Lee and co-workers explores the potential of perovskite nanocrystals as single-photon sources for quantum information technologies [6]. The authors discussed not only their unique photophysical characteristics—such as suppressed blinking and strong light–matter interactions—but also current limitations of these materials, including structural instability, short photonic coherence, iodide instability and difficulties in achieving electrically driven devices. They highlight potential strategies such as surface molecular engineering and epitaxial core–shell structures to overcome these obstacles.

The research article by Tress and colleagues employs transient drift-diffusion simulations to study the behaviour of perovskite LEDs (PeLEDs) under pulsed operation [7]. Their findings reveal that mobile ions play a critical role in shaping the transient electroluminescence (TrEL) response, with injection barriers at the perovskite/transport layer interfaces being a major source of non-radiative recombination. In another research article, Chen, Zhao and co-workers present direct experimental evidence for small polaron formation in Dion–Jacobson-type 2D perovskites, using transient spectroscopy [8]. The study demonstrates how polaron formation enhances spin lifetime by up to 10-fold and produces unusually long polarization response times (∼600 ps), attributed to lattice distortion and two-unit-cell-sized polarons.

Two additional research papers focus on zero-dimensional (0D) organic–inorganic metal halides (OIMHs). Wang, Peng and colleagues use low-temperature magneto-optical spectroscopy to study (Bmpip)₂SnBr₄, resolving the origin of the commonly observed dual-peak emission in 0D-OIMHs [9]. Their findings indicate that the high-energy emission arises from bright excited states, while the low-energy peak originates from mixed triplet-bright and singlet-dark states. Li, Zhang and co-workers investigate the self-recoverable elastico-mechanoluminescence (EML) of [C₁₉H₁₈P]₂MnBr₄, a 0D hybrid with two polymorphs—one piezoelectric and the other non-piezoelectric [10]. They find that only the piezoelectric phase displays EML behaviour, which is attributed to the piezoelectric effect and stress-induced band tilting. The self-recovery is linked to the phase transition from a kinetically stable non-piezoelectric phase to a thermodynamically favourable piezoelectric one.

Together, these contributions highlight the diversity and richness of halide perovskite materials and their derivatives, providing valuable insights into the structure–property relationships and strategies to improve stability, optoelectronic performance and device integration. We hope this Special Topic will inspire further research and innovation in this rapidly evolving field.
